# Cross-breastfeeding and milk donation in Brazil

**DOI:** 10.1590/0102-311XEN082322

**Published:** 2023-09-25

**Authors:** Cristiano Siqueira Boccolini, Neilane Bertoni dos Reis, Dayana Rodrigues Farias, Talita Lelis Berti, Elisa Maria de Aquino Lacerda, Inês Rugani Ribeiro de Castro, Gilberto Kac, Brazilian Consortium on Child Nutrition, Letícia B. Vertulli Carneiro

**Affiliations:** 1 Instituto de Comunicação e Informação Científica e Tecnológica em Saúde, Fundação Oswaldo Cruz, Rio de Janeiro, Brasil.; 2 Divisão de Pesquisa Populacional, Instituto Nacional de Câncer José Alencar Gomes da Silva, Rio de Janeiro, Brasil.; 3 Instituto de Nutrição Josué de Castro, Universidade Federal do Rio de Janeiro, Rio de Janeiro, Brasil.; 4 Instituto de Nutrição, Universidade do Estado do Rio de Janeiro, Rio de Janeiro, Brasil.

**Keywords:** Breastfeeding, Milk Banks, Nutrition, Maternal Health, Aleitamento Materno, Bancos de Leite Humano, Nutrição, Saúde Materna, Lactancia Materna, Bancos de Leche Humana, Nutrición, Salud Materna

## Abstract

The objective of this study was to describe the frequency of cross-breastfeeding, human milk donation to human milk banks and reception of human milk from human milk banks, and to investigate the intersection between cross-breastfeeding and breast milk donation practices. This study used data from the national household-based survey *Brazilian National Survey on Child Nutrition* (ENANI-2019), which collected information from 14,558 children < 5 years old between February 2019 and March 2020. The present study included data from 5,831 biological mothers who reported having breastfed their child < 2 years old at least once and replied questions about cross-breastfeeding, donation and recaption of human milk to human milk banks. Prevalence and 95% confidence intervals (95%CI) were estimated for each stratifier, considering the study complex sample design. Among mothers of children < 2 years old who breastfed their child at least once, 21.1% practiced cross-breastfeeding; breastfeeding another child was more frequent (15.6%) than allowing a child to be breastfed by another woman (11.2%). Among this population, 4.8% of women donated human milk to a human milk bank, and 3.6% reported that their children had received donated human milk. The donation of human milk is a practice recommended by the Brazilian Ministry of Health and has the potential to save thousands of newborns throughout Brazil. In contrast, cross-breastfeeding is contraindicated due to the potential risk of transmitting HIV. There is a need for a broad debate on these practices in Brazil and worldwide.

## Resumo

O objetivo deste estudo foi descrever a frequência de amamentação cruzada, doação de leite humano para bancos de leite humano e recepção de leite humano dos bancos de leite humano, além de investigar a interseção entre práticas de amamentação cruzada e a doação de leite materno. Este estudo utilizou dados do *Estudo Nacional de Alimentação e Nutrição Infantil* (ENANI-2019), uma pesquisa populacional de base domiciliar que coletou informações de 14.558 crianças < 5 anos entre fevereiro de 2019 e março de 2020. Dados de 5.831 mães biológicas que relataram ter amamentado seu filho com menos de dois anos de idade pelo menos uma vez e que responderam às perguntas sobre amamentação cruzada, doação e recepção de leite humano nos bancos de leite humano foram inclusos. Foram estimados as prevalências e os intervalos de 95% de confiança (IC95%) para cada estratificador, considerando o desenho amostral complexo do estudo. Entre as mães de crianças com menos de dois anos que amamentaram o filho pelo menos uma vez, 21,1% praticaram a amamentação cruzada. Amamentar outra criança foi mais frequente (15,6%) do que permitir que a sua criança fosse amamentada por outra mulher (11,2%). Entre essas mulheres, 4,8% doaram leite humano para um bancos de leite humano e 3,6% relataram que seus filhos receberam leite humano doado. A doação de leite humano é uma prática recomendada pelo Ministério da Saúde e tem o potencial de salvar milhares de recém-nascidos em todo o Brasil. Em contraste, a amamentação cruzada é contraindicada devido ao risco potencial de transmissão do HIV. Há necessidade de um amplo debate sobre essas práticas no Brasil e no mundo.

## Resumen

El objetivo de este estudio fue describir la frecuencia de lactancia materna cruzada, la donación de leche humana a los bancos de leche humana y la recepción de leche humana de los bancos de leche humana, además de investigar la intersección entre las prácticas de lactancia materna cruzada y la donación de leche materna. Este estudio utilizó datos del *Estudio Nacional de Alimentación y Nutrición Infantil* (ENANI-2019), una encuesta nacional de hogares que recopiló información de 14.558 niños < 5 años, en el periodo entre febrero de 2019 y marzo de 2020. Se incluyeron datos de 5.831 madres biológicas que reportaron haber amamantado a su hijo < 2 años, al menos una vez, y que respondieron preguntas sobre lactancia cruzada, donación y recepción de leche humana en los bancos de leche humana. Se estimaron prevalencias y los intervalos de 95% de confianza (IC95%) para cada estrato, considerando el diseño muestral complejo del estudio. Entre las madres de niños < 2 años que amamantaron a su hijo al menos una vez, el 21,1% practicaba la lactancia cruzada. Amamantar a otro hijo fue más frecuente (15,6%) que dejar que su hijo sea amamantado por otra mujer (11,2%). Entre estas mujeres, el 4,8% donó leche humana a un bancos de leche humana y el 3,6% informó que sus hijos recibieron leche humana donada. La donación de leche humana es una práctica recomendada por el Ministerio de Salud brasileño y puede salvar muchos recién nacidos en todo Brasil. Por el contrario, la lactancia cruzada está contraindicada debido al potencial riesgo de transmisión del VIH. Es necesario un amplio debate sobre estas prácticas en Brasil y en el mundo.

## Introduction

Breastfeeding another child other than a woman’s child or allowing a child to be breastfed by a woman other than the mother is called cross-breastfeeding or cross-nursing. Although no official definition of cross-breastfeeding was established by the World Health Organization (WHO), United Nations Children’s Fund (UNICEF), or by the Brazilian Ministry of Health, Thorley ^1^ defines it as the sharing of breastfeeding duties among equals. The author distinguishes it from “wet nurse”, who, in a nonreciprocal relationship with the birth mother, breastfeeds a child for another woman, often for financial compensation. Cross-breastfeeding usually occurs between relatives or friends and is understood by mothers as an act of solidarity and altruism ^2^.

The frequency with which cross-breastfeeding occurs in Brazil and worldwide is unknown, and the few Brazilian studies on the topic have reported a 29.4% prevalence among mothers in Rio de Janeiro, 34.5% in Petrópolis (Rio de Janeiro State), and 43.4% in Queimados (Rio de Janeiro State) ^3^ ^,^ ^4^. Cross-breastfeeding has been contraindicated in Brazil since 1996 ^5^ to prevent perinatal transmission of HIV. This recommendation remains valid, and its importance in preventing HTLV-1 and HTLV-2 infection should be emphasized ^5^ ^,^ ^6^.

The Brazilian Network of Human Milk Banks (rBLH Brasil), established by the Brazilian Ministry of Health and the Oswaldo Cruz Foundation (Fiocruz) in 1998, aims to “*promote, protect, and support breastfeeding, collect and distribute human milk with certified quality, and contribute to the reduction in infant mortality*” ^7^. Human milk banks are an option for breastfeeding women to safely donate their milk to another or their own child. By March 2022, there were 222 human milk banks distributed throughout all Brazilian states. In 2019, human milk banks collected 222,696 liters of human milk ^7^.

Although cross-breastfeeding is contraindicated and the Brazilian Ministry of Health recommends donating breast milk to a human milk bank, both practices result in the supply of human milk from a nursing mother to an infant who is not her child. Evaluating the frequency of these practices at the national level and in population subgroups can contribute to formulate health communication strategies and strengthen breastfeeding actions and policies. This study aimed to describe the frequency of cross-breastfeeding and the donation and reception of human milk and investigate the intersection between cross-breastfeeding and practices of breast milk donation.

## Methods

This study used data on breastfeeding practices collected by the *Brazilian National Survey on Child Nutrition* (ENANI-2019). The ENANI-2019 was a national household-based population survey, representative of children < 5 years old, with complex probability sampling, geographic stratification by macroregion, conglomeration by census tracts, and weight calibration. Data were collected from February 2019 to March 2020 by home interviews using a structured questionnaire. The general methodological and sampling aspects can be found elsewhere ^8^ ^,^ ^9^ ^,^ ^10^.

ENANI-2019 collected data from 14,558 children and, this manuscript, data were obtained for 5,831 biological mothers who reported having breastfed their children < 2 years old (< 730 days) at least once. For this subsample, women were asked questions about cross-breastfeeding, donation, and reception of human milk from a human milk bank. For women with more than one child < 2 years old, the questions referred to the youngest child.

To calculate the cross-breastfeeding indicators, two questions were asked: (1) “Since you breastfeed, or when you were breastfeeding ‘children name’, have you ever breastfed another woman’s child?” and (2) “Since you breastfeed, or when you were breastfeeding ‘children name’, have you ever allowed your child to be breastfed by another woman?”. To calculate human milk donation and reception, two other questions were asked: (1) “Since you breastfeed, or when you were breastfeeding ‘children name’, have you ever donated your milk to a human milk bank or a human milk collection point?” and (2) “Since you breastfeed, or when you were breastfeeding ‘children name’, have you ever received milk from a human milk bank?”.

These answers were critically analyzed to identify inconsistencies, missing values, or “do not know” or “did not want to answer” responses ^11^. Automatic imputation method (sequential “hot deck”) were implemented in CSpro and “closest neighbor” using the “knn” function of the VIM package of the R (http://www.r-project.org) were used ^11^ ^,^ ^12^ ^,^ ^13^. The choice of donors considered socioeconomic factors potentially associated with the imputed variables (income quarter of the census tract, age, and maternal education level), seeking donors within the same municipality or, when not possible, within the same macroregion. Thus, all variables used in this study had complete information, except for maternal age, which had ten missing values.

Cross-breastfeeding was evaluated based on four indicators: (1) proportion of mothers of children < 2 years old who breastfed children other than their own; (2) proportion of mothers of children < 2 years old who let another nursing mother breastfeed their youngest child, (3) the occurrence of both practices (allow the child to be breastfed by another woman and breastfed the child of another woman), and (4) the occurrence of at least one of these practices (allow the child to be breastfed by another woman or breastfed the child of another woman), here called total cross-breastfeeding.

Breast milk donation and reception were evaluated using the following indicators: (1) breast milk donation, proportion of mothers of children < 2 years old who donated breast milk while breastfeeding their youngest child; (2) breast milk reception, proportion of mothers of children < 2 years old whose youngest child received donated breast milk; (3) combination of donation and reception, proportion of mothers of children < 2 years old who donated and received breast milk, and (4) proportion of mothers of children < 2 years old who did not donate breast milk nor their children received donated breast milk.

A total of four indicators were calculated for the combination of cross-breastfeeding and human milk donation among mothers of children < 2 years old: (1) proportion of mothers who breastfed another child and who did not donate breast milk; (2) proportion of mothers who breastfed another child and donated breast milk; (3) proportion of mothers who did not breastfeed another child and who donated breast milk; and (4) proportion of mothers who did not breastfeed another child nor donate breast milk. These indicators consider a practice present if the woman reported a practice at least once. Neither the volume of milk donated or received nor the frequency of these practices was considered.

The prevalence and 95% confidence intervals (95%CI) for each indicator were calculated. To identify vulnerable sub-groups, the analysis was stratified according to Brazilian macroregions (North, Northeast, Southeast, South, and Central-West), education level (0-7; 8-10; 11; ≥ 12 years of education); maternal age (< 20 [adolescents]; 20-34; ≥ 35 years old), and the National Wealth Score (IEN), which is a synthetic household index that assesses family socioeconomic conditions. The analyses were stratified into thirds of IEN (poorest, intermediate, and wealthiest households) ^14^. Differences in prevalence were considered statistically significant between categories of stratifiers when there was no overlap of the confidence interval for the point estimates.

The analyses were performed in the software R using the *srvyr* and *survey* packages, considering the sampling plan structure, the weights, and the calibration used to compensate for nonresponses to match the population estimates to the total known population ^11^.

### Ethical considerations

The ENANI-2019 was approved by the Research Ethics Committee of the Clementino Fraga Filho University Hospital of the Federal University of Rio de Janeiro (UFRJ; CAAE n. 89798718.7.0000.5257). Data were collected after a parent or caregiver of the child authorized participation in the study through informed consent form.

## Results

In Brazil, 21.1% of mothers of children < 2 years old who breastfed their child at least once practiced cross-breastfeeding, with the practice of breastfeeding another child being more frequent (15.6%) than allowing the child to be breastfed by another woman (11.2%). These two practices were more prevalent in the North Region (27.8% and 15.8%, respectively) and less prevalent in the South Region (9.3% and 6.9%, respectively); differences were statistically significant. The point estimates indicate that allowing a child to be breastfed by a woman other than the mother was more frequent among women with lower education levels (0-7 years of schooling), with a prevalence twice as high compared with women with higher education levels (≥ 12 years of education) (16.2% and 8.3%, respectively). The concurrence of both practices occurred among 5.7% of these women, with no statistically significant differences based on the stratifiers evaluated. Total cross-breastfeeding (21.1%) was almost three times more frequent in the North Region (34.8%) than in the South Region (12.5%); the difference was statistically significant. Women < 20 years old (35.3%) practiced cross-breastfeeding twice as much as women ≥ 35 years old (17%); the difference was statistically significant (Table 1).

**Table 1:** Prevalence of cross-breastfeeding practices in Brazil and according to sociodemographic characteristics, 2019.

Sociodemographic characteristics	Breastfed children other than their own		Allowed the youngest child to be breastfed by another mother		Both practices *		Total cross-breastfeeding **	
	%	95%CI	%	95%CI	%	95%CI	%	95%CI
Brazil	15.6	13.4; 17.9	11.2	8.8; 13.6	5.7	4.2; 7.2	21.1	18.1; 24.2
Brazilian macroregions								
North	27.8	22.5; 33.0	15.8	10.6; 21.0	8.8	4.3; 13.3	34.8	28.2; 41.3
Northeast	14.9	9.6; 20.3	11.7	6.6; 16.8	6.3	2.5; 10.1	20.3	14.0; 26.6
Southeast	15.1	11.3; 18.9	11.5	7.0; 16.0	5.3	3.1; 7.5	21.3	15.4; 27.1
South	9.3	6.1; 12.4	6.9	4.0; 9.8	3.7	1.6; 5.8	12.5	8.7; 16.3
Central-West	14.4	11.2; 17.6	8.9	6.7; 11.2	4.6	3.2; 6.1	18.7	14.9; 22.4
IEN (tertiles)								
First	15.5	12.9; 18.2	13.1	11.0; 15.2	6.3	4.8; 7.8	22.3	19.6; 25.1
Second	15.9	12.5; 19.4	11.2	8.1; 14.3	5.2	3.0; 7.4	21.9	17.7; 26.2
Third	15.5	11.3; 19.6	9.2	5.0; 13.4	5.6	2.9; 8.3	19.0	13.6; 24.4
Education level of the mother (years of education)								
0-7	17.9	11.9; 23.8	16.2	11.0; 21.3	8.4	4.3; 12.4	25.7	19.4; 32.0
8-10	18.4	14.6; 22.2	14.0	9.8; 18.2	7.1	4.5; 9.8	25.3	20.5; 30.0
11	14.6	11.3; 17.8	8.4	6.1; 10.7	4.3	2.8; 5.8	18.7	14.9; 22.5
≥ 12	12.1	6.9; 17.2	8.3	5.1; 11.6	4.2	1.5; 6.9	16.3	11.3; 21.2
Maternal age (years) ***								
< 20	25.9	18.2; 33.7	21.7	14.7; 28.8	12.4	6.3; 18.5	35.3	27.6; 42.9
20-34	14.8	12.5; 17.0	10.3	8.0; 12.6	5.0	3.6; 6.4	20.0	17.2; 22.9
≥ 35	12.9	7.7; 18.2	8.4	4.6; 12.3	4.4	1.4; 7.5	17.0	11.4; 22.5

95%CI: 95% confidence interval; IEN: National Wealth Score.

Note: prevalence was calculated considering only mothers who breastfed the youngest child younger than 2 years old (< 730 days, n = 5,831).

* Breastfed another child and allowed the child to be breastfed;

** Breastfed another child or allowed the child to be breastfed;

*** The data for ten women were excluded from the analysis due to missing values.

In Brazil, 4.8% of mothers of children < 2 years old who breastfed their child donated breast milk to a human milk bank. The prevalence in the Southeast Region (2.5%) was significantly lower than that in the Central-West (6.9%) and South (7.1%) regions. The prevalence of breast milk donation was higher among mothers in the 1st third of the IEN than among mothers in the last third of this stratifier (5.7% and 3.4%, respectively); however, the difference was not statistically significant. The prevalence of donating milk was higher among mothers < 20 years old than those ≥ 35 years old (8.1% and 3.3%, respectively) (Table 2).

**Table 2:** Prevalence of breast milk donations and breast milk received in Brazil and according to sociodemographic characteristics, 2019.

Sociodemographic characteristics	Breast milk donation		Breast milk reception		Both donation and reception		No donation or reception	
	%	95%CI	%	95%CI	%	95%CI	%	95%CI
Brazil	4.8	3.8; 5.8	3.6	2.7; 4.5	0.8	0.3; 1.2	92.4	91.1; 93.6
Brazilian macroregions								
North	6.1	2.6; 9.5	5.8	3.8; 7.9	1.0	0.0; 2.2	89.1	85.5; 92.7
Northeast	5.7	3.8; 7.5	4.4	2.3; 6.4	1.2	0.0; 2.4	91.2	88.7; 93.7
Southeast	2.5	1.0; 4.1	1.8	0.5; 3.1	0.3	0.0; 0.8	96.0	94.0; 98.0
South	7.1	4.7; 9.5	3.3	1.4; 5.2	0.6	0.2; 1.1	90.2	86.7; 93.7
Central-West	6.9	4.5; 9.3	6.7	5.0; 8.5	1.1	0.6; 1.7	87.5	84.4; 90.6
IEN (tertiles)								
First	5.7	4.4; 6.9	4.7	3.8; 5.6	1.1	0.5; 1.6	90.7	89.2; 92.1
Second	5.3	3.2; 7.4	3.3	1.8; 4.8	0.8	0.0; 1.8	92.2	89.7; 94.8
Third	3.4	1.4; 5.3	2.7	1.2; 4.2	0.3	0.0; 0.9	94.3	91.9; 96.7
Education level of the mother (years of education)								
0-7	4.5	2.5; 6.6	3.3	1.7; 4.9	0.2	0.0; 0.3	92.3	89.7; 94.9
8-10	5.2	3.0; 7.5	4.3	2.5; 6.0	1.6	0.1; 3.1	92.1	89.8; 94.5
11	4.4	3.0; 5.9	3.8	2.3; 5.3	0.6	0.2; 0.9	92.3	90.2; 94.5
≥ 12	5.3	2.5; 8.1	2.7	1.4; 4.0	0.7	0.0; 1.6	92.7	89.9; 95.6
Maternal age (years) *								
< 20	8.1	4.7; 11.5	5.5	2.7; 8.3	1.1	0.0; 2.5	87.4	83.2; 91.7
20-34	4.7	3.5; 5.9	3.4	2.5; 4.3	0.8	0.3; 1.3	92.7	91.4; 94.1
≥ 35	3.3	1.3; 5.3	3.2	1.4; 4.9	0.3	0.1; 0.5	93.9	91.4; 96.4

95%CI: 95% confidence interval; IEN: National Wealth Score.

Note: prevalence was calculated considering only mothers who breastfed the youngest child < 2 years old (< 730 days, n = 5,831).

* The data for ten women were excluded from the analysis due to missing values.

The proportion of women who reported that their child received donated breast milk was 3.6%, with the highest prevalence in the Central-West (6.7%) and North (5.8%) regions and the lowest in the Southeast Region (1.8%); these differences were statistically significant. The point estimate was also higher for mothers belonging to the 1st third of the IEN (4.7%) than those from the last third (2.7%). The concurrence of human milk donation and reception in Brazil was 0.8%, lower in the Southeast Region (0.3%) and higher in the Central-West Region (1.1%); the difference was statistically significant. The proportion of mothers who donated human milk and whose children received human milk was 0.8%, with no differences based on IEN, education level, and maternal age (Table 2).

The practice of breastfeeding another child but not donating breast milk was reported by 13.7% of mothers, being more frequent in the North (24.8%) and less frequent in the South (6.9%). The prevalence of this indicator was higher among mothers < 20 years old (23.5%) than among mothers aged from 20-34 years (12.8%); the difference was statistically significant. The prevalence of mothers that did not bresatfeed another child and donated human milk was 2.9%. The prevalence of breastfeeding a child but not donating milk was higher among mothers belonging to the 1st third of the IEN and those < 20 years old than among mothers in the last third of the IEN and ≥ 35 years old, respectively, with overlapping confidence intervals. Only 1.9% of mothers reported the concomitant practice of breastfeeding another child and donating their milk. No statistically significant differences were found after variable stratification (Table 3).

**Table 3:** Prevalence of cross-breastfeeding and breast milk donations in Brazil and according to sociodemographic characteristics, 2019.

Sociodemographic characteristics	Breastfed and another child and who did not donate		Did not breastfeed another child and donated		Breastfed another child and donated		Did not breastfeed another child and did not donate	
	%	95%CI	%	95%CI	%	95%CI	%	95%CI
Brazil	13.7	11.5; 16.0	2.9	2.2; 3.5	1.9	1.2; 2.6	81.5	79.2; 83.8
Brazilian macroregions								
North	24.8	17.5; 32.1	3.1	1.4; 4.8	2.9	0.1; 5.8	69.1	63.1; 75.2
Northeast	12.4	7.6; 17.2	3.1	1.7; 4.6	2.5	1.1; 4.0	82.0	76.7; 87.3
Southeast	14.0	10.0; 18.1	1.5	0.5; 2.4	1.1	0.1; 2.0	83.4	79.6; 87.2
South	6.9	4.2; 9.7	4.8	3.2; 6.4	2.3	1.1; 3.5	85.9	82.2; 89.7
Central-West	12.7	9.6; 15.9	5.2	3.3; 7.2	1.6	0.8; 2.4	80.4	76.6; 84.2
IEN (tertiles)								
First	13.9	11.3; 16.6	4.1	2.9; 5.2	1.6	1.2; 2.0	80.4	77.6; 83.2
Second	13.4	9.9; 16.8	2.7	1.5; 3.9	2.5	0.9; 4.2	81.3	78.0; 84.6
Third	13.9	9.4; 18.4	1.8	0.6; 3.0	1.6	0.3; 2.8	82.8	78.4; 87.1
Education level of the mother (years of education)								
0-7	16.5	11.3; 21.7	3.2	1.3; 5.1	1.4	0.1; 2.6	79.0	73.0; 84.9
8-10	15.6	12.0; 19.2	2.4	1.3; 3.5	2.8	1.1; 4.5	79.2	75.1; 83.3
11	13.1	9.9; 16.3	3.0	1.8; 4.1	1.5	0.5; 2.4	82.5	79.0; 85.9
≥ 12	9.8	5.5; 14.0	3.0	1.6; 4.4	2.3	0.4; 4.3	84.9	79.6; 90.3
Maternal age (years) *								
< 20	23.5	15.9; 31.2	5.7	2.5; 8.8	2.4	1.0; 3.8	68.4	61.2; 75.6
20-34	12.8	10.4; 15.2	2.7	2.0; 3.4	2.0	1.1; 2.8	82.5	80.1; 84.9
≥ 35	11.6	7.0; 16.2	1.9	0.5; 3.4	1.3	0.0; 2.9	85.1	79.9; 90.4

95%CI: 95% confidence interval; IEN: National Wealth Score.

Note: prevalence was calculated considering only mothers who breastfed the youngest child < 2 years old (< 730 days, n = 5,831).

* The data for ten women were excluded from the analysis due to missing values.

## Discussion

The results of ENANI-2019 showed that approximately one in five mothers practiced cross-breastfeeding, with greater frequency in the North region and among younger mothers and that approximately one in twenty mothers donated their milk to a human milk bank. Both practices involve supplying breast milk from a nursing mother to a child other than her child, with the former practice being contraindicated due to the risk of transmission of etiological agents of diseases ^5^. In turn, donating milk to a human milk bank is a recommended practice and an important intervention for the health of preterm children ^15^. All nursing mothers are potential breast milk donors for human milk banks. It is reasonable to infer that some mothers who breastfeed another child could donate excess milk to a human milk bank if they had the opportunity and were encouraged and supported to do so.

An integrative review by Lima et al. ^16^ on cross-breastfeeding, identified only nine articles using the peer review method in the literature published in Portuguese, English, or Spanish: three in Turkey, one in the United States, and five in Brazil. However, the studies conducted in Turkey and the United States evaluated processes of milk sharing or wet nursing, which, according to the classification proposed by Thorley ^1^, imply a financial relationship (purchase of donated milk or payment of freight or transport for its delivery) or dependence between peers. On the other hand, cross-breastfeeding is an exchange between peers with no apparent social or economic advantage.

Among the studies on cross-breastfeeding conducted in Brazil, von Seehausen et al. ^3^ investigated 695 children younger than 1 year old in Rio de Janeiro in 2013 and observed that cross-breastfeeding occurred in 29.4% of postpartum women who attended primary healthcare units in the city. A study of birth cohorts conducted in Queimados (n = 586) and Petrópolis (n = 732) from 2008 to 2010 showed a prevalence of cross-breastfeeding of 43.4% and 34.5% among children < 6 months, respectively ^4^. In these two studies, the data collection methodology and the categorization of the cross-breastfeeding variable were similar to ENANI-2019. However, the ENANI-2019 population comprised children < 2 years old. Notably, in both Petrópolis and Rio de Janeiro, the practice of cross-breastfeeding was higher among adolescent mothers, similar to that found in ENANI-2019.

A birth cohort study conducted in Rio Branco (Acre State) with 833 children in 2015 showed a prevalence of cross-breastfeeding of 18.6% ^17^. A survey conducted in a philanthropic maternity hospital in Aracajú (Sergipe State) assessed 135 women in 2021 and found a prevalence of cross-breastfeeding of 49.2% ^18^. These studies indicate great variability in this practice in Brazilian municipalities and regions. This finding may be related to cultural factors and differences in the sociodemographic composition of the population.

A definition of cross-breastfeeding is essential to guide discussions on this topic both in national and international scenarios, to plan public health actions and policies addressing this practice, and to measure it in national and local surveys focused on feeding and infant nutrition, and to follow its trend over time. However, the WHO, UNICEF, and the Brazilian Ministry of Health have not yet provided definitions for it.

The only Brazilian study that evaluated the prevalence of human milk donations to human milk banks was conducted in Rio de Janeiro in 2013; 695 pairs of mothers and children were evaluated, and the results indicated that 7.3% of the women donated milk to a human milk bank ^19^. Meneses et al. ^19^ collected data in the primary health care linked to the Brazilian Unified National Health System (SUS), which initiated actions to support breastfeeding and to encourage milk donation; their results regarding milk donations to a human milk bank were approximately 2.5 percentage points higher than those observed in ENANI-2019. These findings allow us to infer that milk donations occur at a prevalence relatively similar to that found in this study, even in favorable environments.

Brazil has the largest human milk bank network worldwide ^20^, with centers in all Federative Units offering human milk with certified quality for preterm infants and newborns at risk ^21^ ^,^ ^22^. Human milk banks are considered a strategic breastfeeding action of the Brazilian National Policy for Integral Child Health Care (PNAISC) ^23^. Every breastfeeding mother has the potential to donate her excess human milk to a human milk bank, and those who practice cross-breastfeeding theoretically have enough human milk for donation. More than one-third of mothers who donated their milk to a human milk bank also practiced cross-breastfeeding (1.9% of 4.8%).

Considering that 4.8% of women donated human milk in 2019, and human milk banks collected 222,696 liters of human milk in the same year ^21^, we can estimate that an increase of 1% in the donation prevalence would represent an increase of 46,395 liters of human milk collected (simulation not shown in the *Results*).

To the best of our knowledge, no study in the academic literature has simultaneously evaluated cross-breastfeeding and human milk donation in Brazil or any other country. The findings of ENANI-2019 can serve as a baseline for monitoring these practices and promoting public health policies, actions, and programs focused on the subject.

As a limitation of the study, it was not possible to evaluate the frequency and duration of cross-breastfeeding or the relationship of the mother who breastfed with the family of the child who received the milk: if they knew each other - as friends, relatives, or neighbors - or not. We also did not evaluate the frequency and amount of human milk donation nor how long breastfeeding mothers donated their excess milk. The same can be applied to human milk reception, with no information about the volume and frequency received nor how long the infants received donated human milk.

Due to the phenomenon’s complexity and lack of an official definition, we suggest conducting validation studies to improve questions addressing cross-breastfeeding, bringing more precise estimates of this practice. Regarding human milk reception, it is unknown if mothers were fully aware of the content of milk supplementation offered to their infants. There may have been confusion about whether infant formulas or human milk were given, generating the possibility of overestimating or underestimating this practice.

ENANI-2019 was designed to identify infant feeding and nutrition patterns in Brazil and macroregions, not allowing local analyses. Therefore, the prevalence of human milk donation might be interpreted as the percentage of breastfeeding mothers in Brazil that accessed a human milk bank and donated their excess milk. Considering that the donation of human milk to a human milk bank depends on, among other factors, the existence of a human milk bank in the city or region where the person lives and their access to it, the prevalence of donation may be underestimated for municipalities that have human milk banks and overestimated for those who do not have such service. Nevertheless, the milk donation indicator was included in the list of collected data, allowing a population estimate of this practice in Brazil and monitoring its trend in future studies.

To understand the potential bias resulting from the presence or absence of a human milk bank in municipalities altering the estimates of the prevalence of human milk donation, a crossover of the information from municipalities with human milk banks ^22^ was performed with the 123 municipalities that composed the ENANI-2019 sample. A total of 65 municipalities in the ENANI-2019 sample (55.8%) had at least one operating human milk bank (data not shown).

The main strength of ENANI-2019 was the inclusion, for the first time in a survey of national representativeness, of questions that allowed measuring cross-breastfeeding and human milk donation to human milk banks, giving visibility to these practices and allowing them to be points on the agenda for a broad debate with society, with local and national policy makers, and with international organizations.

The donation of human milk is a practice recommended by the Brazilian Ministry of Health. This practice has the potential to save thousands of newborns throughout Brazil via the supply of pasteurized human milk certified by the human milk bank. In contrast, cross-breastfeeding is contraindicated due to the potential risk of transmitting HIV. The significant frequency of cross-breastfeeding practices indicates the presence of potential human milk donors for human milk banks throughout Brazil.

There should be a broad debate with policy makers, the academic community, and families on cross-breastfeeding. Local and global agents should define the terminology and determine metrics. Finally, communication campaigns encouraging human milk donation to human milk banks should be strengthened, with guidelines aimed at vulnerable mothers and those who practice cross-breastfeeding.
